# Regulation of ABCB1 activity by microRNA-200c and microRNA-203a in breast cancer cells: the quest for microRNAs’ involvement in cancer drug resistance

**DOI:** 10.20517/cdr.2019.24

**Published:** 2019-09-19

**Authors:** Ana Armada, Bruno Costa Gomes, Miguel Viveiros, José Rueff, António Sebastião Rodrigues

**Affiliations:** ^1^Global Health and Tropical Medicine, Instituto de Higiene e Medicina Tropical, Universidade NOVA de Lisboa, Rua da Junqueira 100, Lisbon 1349-008, Portugal.; ^2^Centre for Toxicogenomics and Human Health, Genetics, Oncology and Human Toxicology, NOVA Medical School/Faculdade de Ciências Médicas, Universidade Nova de Lisboa, Rua Câmara Pestana 6, Lisbon, Lisbon 1150-008, Portugal.

**Keywords:** *ABCB1*, drug resistance, drug extrusion, breast cancer, miR2003, miR203

## Abstract

**Aim:** ABCB1 is a major player in cancer drug resistance. The purpose of this study was to functionally assess the regulation of ABCB1 activity in a doxorubicin-resistant breast cancer cell line by miR-200c and miR-203.

**Methods:** Human breast carcinoma cell lines MCF-7 (Doxorubicin-sensitive and not expressing ABCB1) and KCR (Doxorubicin-resistant and expressing ABCB1) were used to evaluate the expression levels of miR-200c and miR-203 by Real-time quantitative PCR (RT-qPCR). The effects of transient ectopic expression of miRNA-200c and miR-203 on the expression of ABCB1 in KCR and MCF-7 cells was verified by RT-qPCR and Western Blot. The extrusion activity of the ABCB1 pump was analyzed by fluorescence microscopy and flow cytometry through fluorescence substrate retention assays (DiOC_2_) in the presence and absence of the ABCB1 inhibitor verapamil.

**Results:** RT-qPCR results indicated a 100,000-fold increase in ABCB1 mRNA expression levels in KCR cells compared to MCF-7 cells, and is inversely correlated with the expression of miR-203 and miR-200c. The insertion of miR-200c and miR-203 led to a higher retention of DiOC_2_ within KCR cells, and slightly reduced the protein levels of ABCB1 in KCR cells, although the high initial expression of ABCB1 masked the reduction in protein levels. The increased intracellular accumulation of the fluorescent due DiOC_2_ in the presence of the ABCB1 inhibitor verapamil correlated with the inhibition caused by miR-203 and miR-200c in transfected cells.

**Conclusion:** The present study demonstrates that miR-200c and miR-203 exert a negative modulating effect on the activity of ABCB1 associated with doxorubicin resistance.

## Introduction

Breast cancer (BC) is the most common cancer in women. Data reported in 2018 by WHO indicated about 2.1 million new cases worldwide (http://gco.iarc.fr/today/home). Depending on type and staging, BC treatment can be local or systemic. Systemic therapy usually includes a combination of 2 or 3 chemotherapy drugs such as anthracyclines (doxorubicin and epirubicin), taxanes (paclitaxel, docetaxel), 5-fluorouracil, cyclophosphamide and carboplatin; hormone therapy (tamoxifen, fulvestrant, aromatase inhibitors); and targeted therapy (trastuzumab, lapatinib). Unfortunately, most patients treated with these drugs eventually develop resistance, often leading to enhanced disease progression and death^[1]^. Drug resistance is a common manifestation of cancer and is a major factor in the failure of many forms of chemotherapy. Up to 30% of women diagnosed with early-stage BC will eventually progress to metastatic breast cancer (MBC)^[2]^, while cancer drug resistance (CDR) is deemed to be the cause of treatment failure in over 90% of patients with MBC.

Epithelial-mesenchymal transition (EMT) is an adaptation process where cells lose their epithelial characteristics and acquire a mesenchymal phenotype which is associated with tumour progression, metastasis development and drug resistance^[3,4]^. The metastatic cells that have undergone EMT usually exhibit a cancer stem cell-like phenotype normally resistant to chemotherapy due to deregulation of several genes, including the overexpression of ATP-dependent efflux pumps (ABC-transporter proteins), which promotes the efflux of a broad range of anticancer drugs through the cellular membrane^[5]^. In healthy tissues, the presence of this transporter associated with bio-transformation pathways is widely distributed, having a role as “first responder” to compounds that passively cross the membranes, protecting cells from the intracellular accumulation of toxic products. Under drug pressure, cells undergo genetically-mediated adaptations by using various molecular mechanisms, such as the activation of survival signaling pathways and/or an increase in drug efflux transporters. Higher expression of ABC transporters is found in breast, colon, kidney, pancreas or liver tumours. Tumours originated from other tissues may evolve to form oligoclones with genetic heterogeneity acquiring resistance and displaying higher levels of ABC efflux transporters.

ABCB1 (P-glycoprotein or P-gp, MDR1), MRP1 and BCRP proteins are the most prevalent ABC proteins in BC, having the capability to confer multi-drug resistance (MDR). These proteins are part of a complex network of cellular/tissue features which promotes the development of drug resistance in cancer cells^[6]^. Overexpression of ABCB1 is commonly the first mechanism of resistance, preceding the development of other mechanisms like an increase of drug metabolism, mutation of drug targets, activation of DNA repair mechanisms, suppression of apoptosis and induction of EMT, as cells proliferate and adapt to drug regimens^[7]^.

Approximately 50% of human cancer cells express ABCB1 at an adequate amount to confer CDR. Moreover, ABCB1, which is the most prevalent transporter associated with a breast CDR phenotype, confers resistance to a broad range of anticancer drugs, including chemotherapeutics that interfere with DNA (genotoxic drugs), like alkylating agents, anthracyclines, platinum drugs and cytostatic compounds^[8]^, such as taxanes, or vinca alkaloids that disrupt cellular microtubules^[9,10]^. To that set of anti-neoplastic drugs, the newer generation of targeted biological agents should still be added, including in particular the tyrosine kinase inhibitors.

There is an urgent need for the development of therapeutic strategies, in order to reverse CDR. Several strategies to overcome drug resistance by regulating the activity of ABCB1 have been widely explored, especially efflux pump inhibitors in combination with anticancer drugs. However, because of the intrinsic efflux activity present in healthy tissues, clinical trials with ABCB1 modulators have shown disappointing results due to the severe side effects observed^[11-13]^.

Given that the expression of ABCB1 is also under control of epigenomic regulation by microRNAs, another strategy to overcome CDR mediated by ABCB1 is to silence the activity of ABCB1 efflux pumps or reduce its expression using these non-coding RNAs^[14]^. MicroRNAs (miRNAs, miRs) are small noncoding RNAs that regulate gene expression by post-transcriptionally targeting the 3’-UTR region of mRNA. They act as master regulators of protein expression by blocking translation and forming a circuitry of epigenomic gene expression^[4]^. Due to their small size (22-25 nucleotides), miRNAs can bind to several different mRNAs, while the same mRNA can be targeted by several miRNAs. The extent of complementarity of the miRNA recognition sequence (seed region) with its target mRNA determines mRNA cleavage with subsequent degradation, when there is a perfect match, or translational repression if the interaction is partially complementary.

The relevance of miRNAs in regulating the expression of ABC transporters in CDR has been recently reviewed^[15,16]^ specifically the expression pattern of miRNAs seems to have a critical role in DR^[17]^. In fact, several miRNAs have been reported to have a direct or indirect role in the regulation of the expression^[15,18,19]^ or activity^[20]^ of ABCB1 in DR in different cancers.

In particular, miR-200c and miR-203 have been linked to drug resistance in different tumours^[21-25]^ and also to EMT transition^[15,26-28]^. Recent studies conducted by us have shown that there is a significant downregulation of miR-203 and miR-200c with increased stage in invasive lobular breast carcinomas^[29]^. In addition, down-regulation of miR-200c was also observed in the MCF-7 breast cancer cell line resistant to doxorubicin, also being associated with a poor chemotherapy response in human breast cancer patients in part by a possible upregulation of ABCB1 expression^[30]^.

The identification of target genes of specific miRNAs has been essentially carried out by bioinformatics, though the actual identification of target genes depends on the expression profiles of both target and miRNA in the tissue or cell under study. Hence, due to the high number of miRNAs identified, relatively few targets have been identified. This is mainly due to the difficulty in functionally identifying these targets taking into account the complexity of gene-miRNA interaction networks. Furthermore, different miRNA-target prediction algorithms predict targets with different techniques and criteria including base pairing, target accessibility and evolutionary conservation of target site. Each algorithm produces widely different lists of predictions with significant false-positive and false-negative rates. Thus, functional assays may shed light on the biological consequences of manipulating *in vitro* the expression of specific miRNAs, constituting an initial approach which can streamline the identification process.

Accordingly, we have functionally assessed the role of miR-200c and miR-203a in the modulation of drug efflux using a doxorubicin resistant breast cancer cell line and DiOC_2_ as a specific fluorescence substrate of this transporter. These cells constitutively overexpress ABCB1 gene and thus display ideal characteristics to study DR^[31,32]^.

## Methods

### Reagents

All reagents for cell culture and verapamil (VP) were purchased from Sigma-Aldrich. Doxorubicin hydrochloride (DOX) was obtained by TEVA (TEVA 2 mg/mL; Pharmachemie B.V. Netherlands), DiOC_2_ (2 mg/mL in DMSO) was obtained from Molecular Probes^TM^. VP stock solution was prepared in water (10 mM), 0.22 µm filter sterilized and stored in aliquots at -20 °C.

### Cell culture

Human breast adenocarcinoma cell lines MCF-7 and its doxorubicin-resistant subline KCR, were kindly provided by Professor Joseph Molnar, Szeged Foundation for Cancer Research, Hungary^[31]^. MCF-7 cell line exhibits some features of differentiated mammary epithelium and KCR cell line exhibits epithelial-mesenchymal transition gene expression pattern^[33]^. MCF-7 and KCR were cultured in Minimal Essential Medium supplemented with 10% (v/v) heat inactivated fetal bovine serum, 1.5 g × l^-1^ sodium bicarbonate, 1 mM sodium pyruvate, 0.1 mM nonessential aminoacids (Gibco), 1% streptavidin-penicillin (100,000 unit’s penicillin and 10 mg streptomycin per ml, Gibco). KCR cells were cultured in the presence of 1 µM of DOX every 3 passages in order to maintain the resistant phenotype. All cell lines were incubated at 37 ºC in a humidified 5% CO_2_ chamber. Culture medium was replaced three times a week and subcultured by trypsinization (0.25% trypsin in media without serum, Gibco) when confluence reaches approximately 80%.

### Ectopic expression and inhibition of miR-203 and miR-200c

Mimetic miRNAs (Pre-miR^TM^ miRNA Precursor hsa-miR-200c-3p # PM11714 and hsa-miR-203a # PM10152; Life Technologies) and miRNAs inhibitors (Anti-miR^TM^ miRNA Inhibitor hsa-miR-200c-3p # AM11714 and hsa-miR-203a-3p # AM10152; Life Technologies) were transfected in KCR and MCF-7, respectively. To simplify, miR-203a will be represented as miR-203.

Negative controls (NC) were Pre-miR^TM^ miRNA Precursor Negative Control #1 (Life Technologies # AM17110) and Anti-miR^TM^ miRNA Inhibitor Negative Control #1 (Life Technologies # AM17010). These negative controls are oligonucleotides similar to miRNA precursors and miRNA inhibitors but without any biological effect.

The transfection complex (oligo + transfection agent) was prepared in EMEM without any supplementation and incubated at room temperature for 15 min. After this time, the transfection complex was added to the cells that already had complete culture medium in a proportion of 1:1. Adherent cells were transfected with a mixture of 0.3 % (v/v) FuGENE HD transfection reagent (Promega) with mimic/anti-miR-203a and mimic/anti-miR-200c (Life Technologies) or non-specific pre/anti-miRNA controls at a final concentration of 30 nM and 50 nM for KCR/MCF-7 respectively, for 24 h, 48 h and 72 h, at 37 ºC, according to the manufacturers' recommendations.

After the incubation period, functional analysis was performed and total RNAs and proteins were purified.

### Immunofluorescence

Cells were washed twice with PBS and resuspended in a small amount of PBS. Cells in suspension were spotted on a multi-well slide and left to dry before being fixed with ice-cold acetone for 10 min. Cells were blocked with 3% BSA in PBS for 1 h and then incubated with anti-human ABCB1 (dilution 1:200) primary antibody (D11: #sc-55510, Santa Cruz Biotechnology) for another hour at 37 ºC in a humid chamber followed by incubation with FITC-conjugated antibody. Slides were washed twice with PBS, counter-stained with DAPI, following the protocol previously described^[34]^. Coverslips were mounted on the slides and imaged using a Nikon DS-Ri1 camera installed in Nikon eclipse 80i microscope using NIS-Elements BR 3.3 software. Fluorescence emissions were acquired using specific filters set for DAPI (laser 405 nm/BP 420-480 nm), FITC dyes (ex 495 nm/BP 515 nm).

### Evaluation of ABCB1 activity by fluorescence microscopy

Cells were seeded on sterilized glass coverslips in 6-well culture plates (for microscopy fluorescence assays) at a density of 2.5 × 10^5^ cells/mL (24 h, 48 h transfection assay) and 10^5^ cells/mL (72 h transfection assay) and allowed to grow overnight at 37 ºC. The following day, ABCB1 efflux pump activity of transfected cells was evaluated as previously described^[35]^. Briefly, after the transfection time (24 h, 48 h and 72 h), medium was removed from coverslips and fresh medium with and without 10 µM VP was added to each duplicated experiment well and incubated for 30 min before addition of 1 μg/mL of DiOC_2_ for 1 h at 37 ºC. Subsequently, medium was removed, and dye free medium was added with the presence or absence of VP for another 1 h. Slides were rinsed twice in PBS and then cells were examined using a fluorescence microscopy. Cells were examined at 485 nm excitation laser and 530/30 nm emission filter (green fluorescence).

### Quantification of cellular DiOC_2_ retention by flow cytometry

Microscopy-based cell imaging can be used for the rapid evaluation of the efflux activity using a low number of cells, however quantification is less accurate. Therefore intracellular accumulation of DiOC_2_ was quantified in parallel by flow cytometry using the same conditions used in fluorescenc microscopy. After 24 h of transfection, KCR cells were seeded on duplicated T25 flasks (with scramble NC, mimic-miR-203 and mimic-miR-200c), at a density of 2.5 × 10^5^ cells/mL and incubated for 48 h at 37 ºC in a humidified atmosphere of 5 % CO_2_. Then, cells were harvested using trypsin, resuspended in complete medium and viable cells quantified by direct microscopy, using a Neubauer-chamber and trypan blue exclusion dye. Cells were placed in Eppendorf tubes at a concentration of 5 × 10^5^ cells/mL and pre-incubated in medium (control cells) or treated with 10 µM VP, for 30 min at 37 ºC and DiOC_2_ (1 μg/mL) was added and cells were incubated for 1 h at 37 ºC^[35]^. To keep efflux pumps inhibited, cells were then resuspended in 500 µL of fresh medium in the presence and absence of VP and incubated for another hour. Cells were washed twice with cold PBS, spin down, resuspended in 500 µL cold PBS and fluorescence retention was measured by flow cytometry, using CytoFlex flow cytometer (Beckman Coulter) at an excitation wavelength of 488 nm and emission recorded in 525/40 BP (FITC-A channel). Data was collected for at least 10,000 events per sample and analysed using FlowJo v10 software (Tree Star, Inc., Ashland, OR).

**Figure 1:**
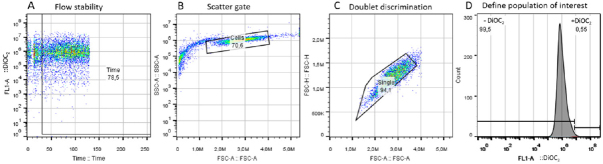
Gating strategy used to select the cell population of interest. (A): flow stability; (B): scatter gate; (C): doublet discrimination; (D): representative histogram of positive and negative fluorescent regions

In order to avoid questionable events, a FITC-A *vs.* time plot parameter was used [Figure 1A]. The area exhibiting a poor flow was excluded from areas of good flow by time gating. Forward scatter area (FSC-A) *vs.* side scatter-area (SSC-A) density plot was used to remove debris and pyknotic cells in the lower left-hand portion of the plot as well as the very large (off scale) debris found in the upper right-hand portion [Figure 1B]. Breast cancer cells (KCR) population was identified based on FSC-A *vs.* SSC-A gate. Singlet gate was used to define the non-clumping cells based on pulse geometry forward scatter height (FSC-H) *vs.* FSC-A, eliminating the doublets [Figure 1C]. Then, gated cells corresponding to viable KCR cells was represented on one-parameter histogram by plotting FL1-A/DiOC_2_ *vs.* the number of events [Figure 1D]. Results were expressed by the median of fluorescence intensity. To estimate the DiOC_2_ retention of untreated transfected cells, the median of fluorescence intensities were calculated according to the following equation:

*Median fluorescence* (*normalized*) = [*miRNA*/(*Average NC*)] × 100;

miRNA is defined as the mean of median of fluorescence intensity retained by KCR transfected cells (miR-NC, miR-200c, miR-203) and average NC is the mean of all the median of fluorescence intensity retained by the negative control transfected cells. To access VP activity on miRNA transfected cells, DiOC_2_ retention was estimated accordintly to the following equation:

*Median fluorescence* (*normalized*) = [(*miRNA + VP*)/(*Average miRNA*) × 100.

miRNA + VP is defined as the mean of median of fluorescence intensity retained by KCR transfected cells (miR-NC, miR-200c, miR-203) treated with verapamil.

### Nucleic acid purification

Total RNA and miRNAs from cell lines were purified with All Prep DNA/RNA Mini kit (Qiagen # 80204) and RNeasy MinElute Cleanup Kit (Qiagen # 74204), according to the manufacture. Briefly, 6 × 10^6^ cells were harvested and lysed in 700 µL of RLT plus buffer and loaded into an AllPrep DNA spin column and centrifuged for 30 s at ≥ 8000 × *g*. The flow-through was used for RNA purification and the DNA spin column stored at room temperature until further use. One volume of 70% ethanol was added to the flow-through and mixed by pipetting. Up to 700 µL of the sample, was loaded into a RNeasy spin column and centrifuged for 30 s at ≥ 8000 × *g*. The flow-through was stored at room temperature for later use for miRNAs purification. RNeasy spin column was washed by adding 700 µL of RW1 buffer and centrifuged for 30 s at ≥ 8000 × *g*. The flow-through was discarded. A second wash with 500 µL of RPE buffer was done, followed by a centrifugation for 30 s at ≥ 8000 × *g*. The flow-through was also discarded. A third wash with 500 µL of RPE buffer was done, followed by a centrifugation for 2 min at ≥ 8000 × *g*. The flow-through was again discarded. Next, 30 µL of nuclease-free water was added directly to the spin column membrane and centrifuged for 1 min at ≥ 8000 × *g* to elute the RNA. This RNA was then stored at -80 °C until further use. One volume of 100% ethanol was added to the flow-through previously stored for miRNAs purification. This mixture was then loaded into a RNeasy MinElute Cleanup column and centrifuged for 30 s at ≥ 8000 × *g*. The flow-through was discarded. A wash was done by adding 500 µL of RPE buffer and centrifuged for 30 s at ≥ 8000 × *g* and discarding the flow-through. A second wash with 500 µL of 80% ethanol was done and centrifuged for 2 min at ≥ 8000 × *g*. The flow-through was discarded and a centrifugation for 5 min at full speed was done in order to dry the filter. Next, 14 µL of nuclease-free water was added to the column and centrifuged for 1 min at maximum speed. The miRNAs were then stored at -80 °C until further use. All samples were quantified using a NanoDrop^TM^ spectrophotometer.

### Reverse transcription qPCR for miRNA

The relative expression of miRNA was quantified by Reverse transcription qPCR (RT-qPCR) by using Universal cDNA synthesis kit II and ExiLENT SYBR® Green master mix from Exiqon in a real time PCR 7300 system, accordantly to the protocol described by the manufacturer and described previously^[36]^. The primers used were: hsa-miR-203a (Exiqon, LNA^TM^ PCR primer set # 204285); hsa-miR-200c-3p (Exiqon, LNA^TM^ PCR primer set # 204482) and as endogenous control U6 snRNA (Exiqon, PCR primer set # 203907).

This methodology was performed to detect miR-200c and miR-203 expression levels in MCF-7 and KCR cell lines after ectopic inhibition or over-expression of both miRNAs. The relative amount of miRNA was normalized with the internal control U6 snRNA using the equation 2^ΔCT^, where ΔCT = CT miRNA - CT U6.

### ABCB1 protein expression by Western Blot

Forty-eight hours after transfection with mimics of miR-203 and miR-200c, cells were harvested, washed with PBS and membrane proteins were isolated by using Mem-PER^TM^ Membrane Protein Extraction Kit (Thermofisher) following the manufacture’s protocol. Proteins were quantified by the Bradford assay (Bio-Rad Laboratories) and approximately 3 µg of protein from each sample was mixed with equal amounts of Laemmli sample buffer (Bio-Rad Laboratories) and boiled before being separated by electrophoresis precast gels (4%-20% Mini-PROTEAN® TGX^TM^ Precast Protein Gels, Bio-Rad #4561093S). Proteins were transferred onto polyvinylidene difluoride membranes, according to the protocol previously described^[37]^. Blots were prepared using the WesternDot^TM^ 625 Goat anti-Mouse Western Blot Kit (#W10132) and probed with anti-human ABCB1 (dilution 1:1000) (D11: # sc-55510, Santa Cruz Biotechnology) and anti-β-actin primary antibodies (Santa Cruz # sc-47778).

The immunoblots were visualized under ultra-violet light and the chemiluminescence signal was captured by ChemiDoc^TM^ Touch Imaging System (BioRad), accordantly to the protocol described by the manufacture (Invitrogen).

### Real-time RT-qPCR quantification of mRNA

Quantification of ABCB1 mRNA was carried out by reverse-transcriptase quantitative polymerase chain reaction (RT-qPCR). Total RNA was extracted as previously described and purity ratios 260/280 and 260/230 were determined by using nanodrop spectrophotometer. Single-stranded cDNA was synthesized from 1µg of total RNA using the High Capacity cDNA Reverse Transcription Kits (Applied Biosystems) in a final reaction volume of 20 µL according to the manufacturer’s instructions. The reaction mixture was then used as a template in a TaqMan Fast real-time quantitative PCR assay using Taqman Universal PCR Master Mix and the 7,300 Real-Time PCR System (Applied Biosystems). The predeveloped Taq-man assays (Assay-on-Demand products from Applied Biosystems) were ABCB1, Hs00184491_m1 and the human GAPDH, 4352934E as the reference gene. All PCR reactions were done in a total volume of 10 µL by using TaqMan® Universal Master Mix II, ROX as an internal reference dye and TaqMan DNA polymerase. Template controls and reverse transcriptase controls (RT negative) for each cDNA synthesis were included.

Thermal cycler conditions were 50 ºC for 2 min; 95 ºC for 10 min followed by 40 cycles at 95 ºC for 15 s and at 60 ºC for 1 min. The mean values of the triplicate RT-qPCR reactions for each assay were normalized with the expression values for each gene. Relative expression of ABCB1 was performed by the comparative 2^-(^ΔΔ^Ct)^ method using the expression of GAPDH in the cDNA by using the standard curve method described by the manufacturer (Applied Biosystems).

### Statistical analyses

Significant differences were determined using the non-parametric Wilcoxon test for two paired samples (GraphPad Prism 6.0, GraphPad Software, Inc, USA). A significance level of 5% (*P* < 0.05) was used to evaluate statistical significance. The mean and standard error of at least three independent experiments and triplicate samples are represented by bar-charts.

## Results

### ABCB1 is only expressed in the doxorubicin resistant breast cancer cell line and is inversely correlated with the expression of miR-203 and miR-200c

**Figure 2:**
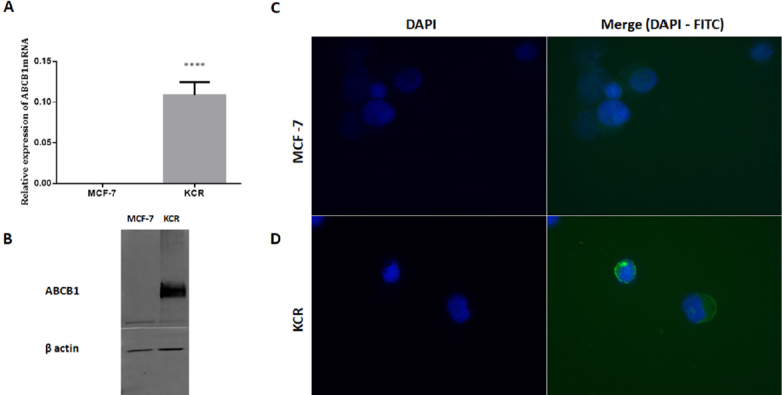
Expression levels of ABCB1 in breast cancer cell lines (MCF-7 and KCR). ABCB1 mRNA levels were analysed by RT-qPCR and the average fold change of ABCB1 gene expression was calculated using 2^-(ΔΔCt)^ method (A). ABCB1 was identified by immunoblotting using β-actin as an internal control (B) Localization of ABCB1 expression was performed in cells incubated with anti-human ABCB1-FITC-conjugated and counter-stained with DAPI. Cells were observed by fluorescence microscopy and images were acquired (C). DNA nuclear - blue; ABCB1 - green. **** *P* < 0.001

ABCB1 is only expressed in the doxorubicin resistant breast cancer cell line and is inversely correlated with the expression of miR-203 and miR-200c The KCR resistant subline expresses both ABCB1 and ABCC1 (Multidrug resistance-associated protein, MRP-1) genes and its parental cell line MCF-7 only expresses MRP-1 (Joseph Molnar, personal communication). By RT-qPCR, the KCR cell line displayed a 100,000-fold increase in ABCB1 mRNA expression levels compared to MCF-7 cells [Figure 2A]. Expression levels of ABCB1 efflux pumps was also significantly up-regulated in KCR cells when analysed by immunofluorescence and western blotting, whereas in MCF-7 ABCB1 was not detectable [Figure 2B, C].

In order to evaluate the role of miR-203 and miR-200c in the MDR phenotype, the basal levels of miR-203 and miR-200c in MCF-7 and KCR cell lines were assessed by RT-qPCR.

**Figure 3:**
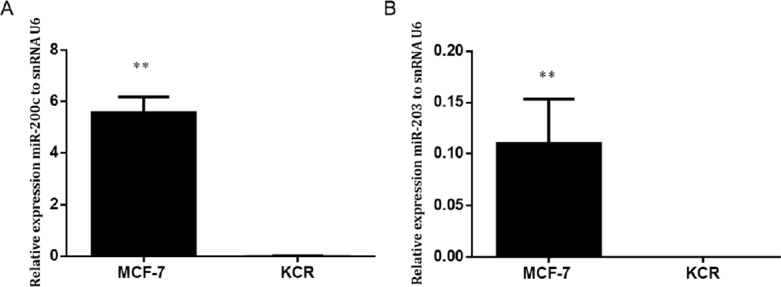
Expression levels of miR-200c and miR-203 in MCF-7 and KCR cell lines. miR-200c (A) and miR-203 (B) was assessed by RT-qPCR and values are represented as the mean relative expression of three independent experiments and three replicates per sample normalized to U6 snRNA ± standard deviation. ** *P* < 0.01

Gene expression level of miR-203 and miR-200c in the KCR resistant cell line was significantly down-regulated, more than 50 fold-change, compared with the parental wild-type MCF-7 where the expression of ABCB1 was not detected [Figure 3]. These results suggest that miR-200c and miR-203 expression have a role in the doxorubicin resistant phenotype mediated by ABCB1 efflux pumps.

In order to assess the role of miRNA-203 in the regulation of ABCB1 expression in drug resistance, we proceeded to transiently transfect mimics and antagomirs of miR-203 and miR-200c in KCR and MCF-7 cell lines respectively, and analysed the efflux activity of ABCB1 pumps in living cells using a ABCB1 specific fluorescent substrate.

**Figure 4:**
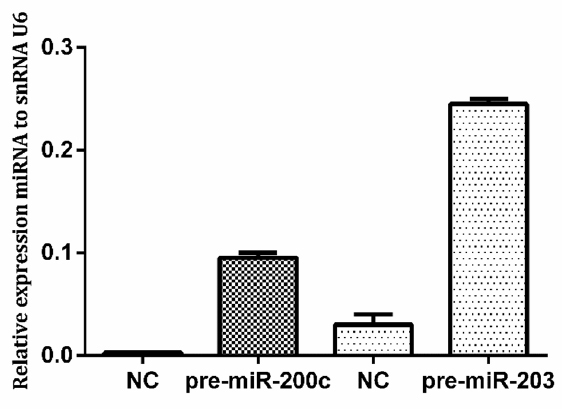
Expression of miR-200c and miR-203 in KCR cells after transfection. Cells were transfected with pre-miR-200c and pre-miR-203 precursors using FUGENE HD and miRNA levels were upregulated compared with control cells (NC). NC: KCR cells transfected with negative control miRNA. Results are represented by mean values of two independent experiments ± standard deviation

Expression of miR-200c and miR-203 after transfection with pre-miR-200c and pre-miR-203 in the KCR cell line was confimed by RT-PCR [Figure 4].

### Mimic-miR-203 and miR-200c impaired the activity of ABCB1 in KCR cells

The results obtained suggest that these microRNAs may play a role in the regulation of expression of the ABCB1 transporter. Thus, to understand whether miR-203 is involved in the regulation of ABCB1, the transport activity of the ABCB1 in miRNA-transfected cells was evaluated by indirectly measuring the intracellular accumulation of the ABCB1 specific fluorescent substrate DiOC_2_, according to previously described techniques^[35]^.

KCR cells, which have downregulation of miR-203 and miR-200c, and MCF-7 which have upregulation of miR-203 and miR-200c, were transfected, respectively, with mimics/antagomiRs of miR-203, miR-200c and recommended mimic-miR and anti-miR precursors (NC) without any biological function, as negative controls, for 24 h, 48 h and 72 h. Transfected cells were then analysed for the ability to retain DiOC_2_ in the presence and absence of VP.

**Figure 5:**
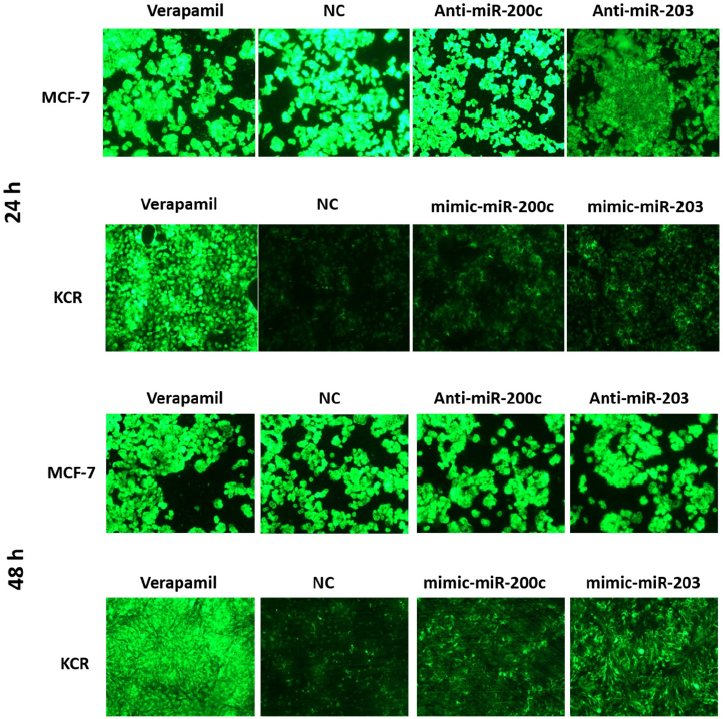
DiOC_2_ accumulation in KCR and MCF-7 cells. KCR cells were transiently transfected with mimic-miR-NC, mimic-miR-200c and mimic-miR-203. After 24 h and 48 h of transfection cells were observed under a fluorescent microscope (× 100 magnification) and images were acquired. In parallel, cells treated with verapamil were also evaluated. Green colour reflects the accumulation of DiOC_2_ inside the cell

Fluorescent microscopy revealed no appreciable differences in the accumulation of DiOC_2_ in MCF-7 cells. However, after 48 h of transfection, KCR cells showed an increase in the accumulation of DiOC_2_ [Figure 5]. This period after transfection was chosen to quantify the intracellular accumulation of DiOC_2_ fluorescence by flow cytometry.

**Figure 6:**
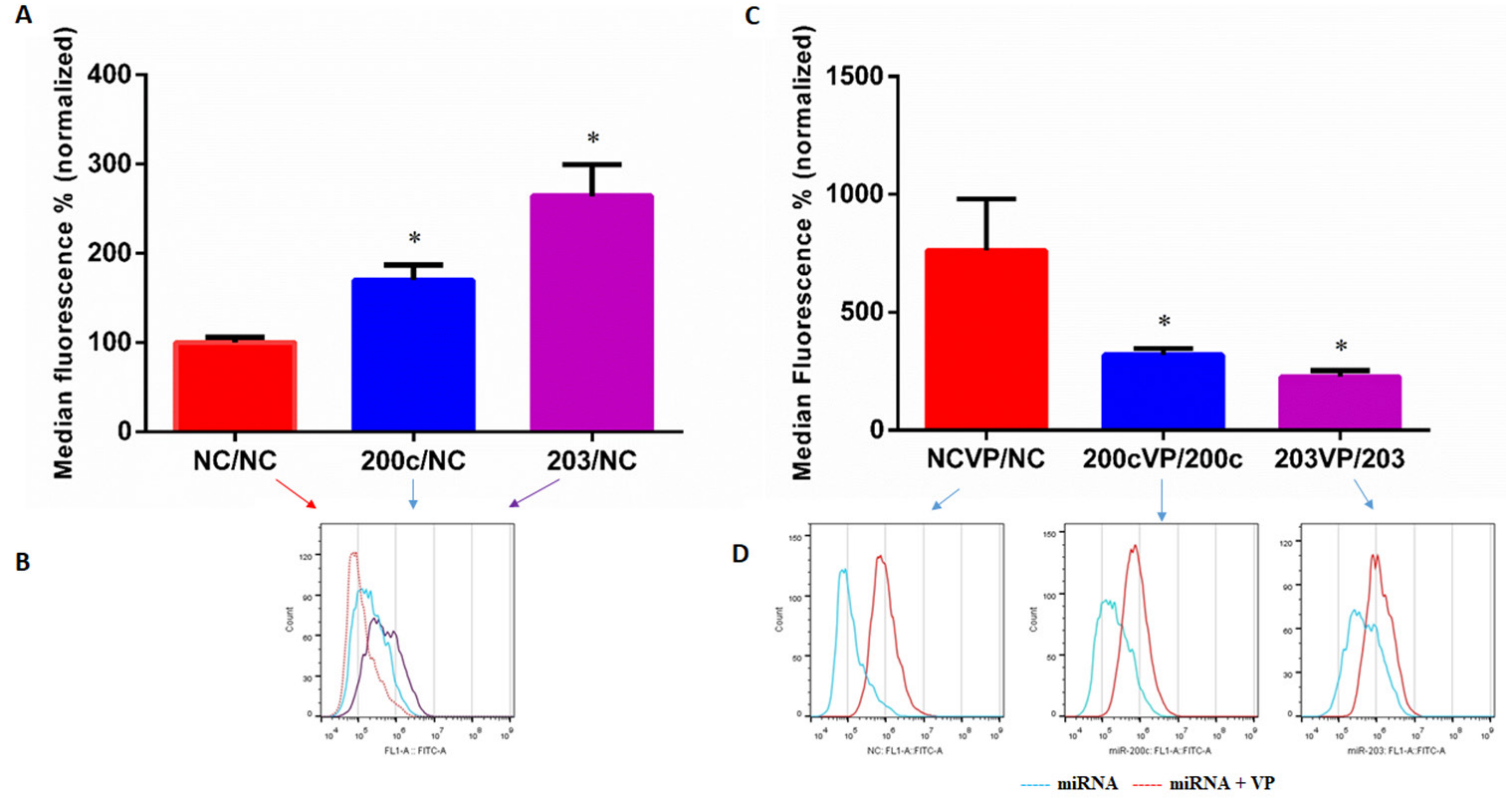
Levels of DiOC2 accumulation in KCR transfected cells. DIOC2 accumulation in KCR cells transfected for 48 h with mimic-miR-negative control (NC), mimic-miR-200c and mimic-miR-203, and treated with verapamil (VP) (C, D), were evaluated by flow cytometry analysis.In parallel, non treated cells were also evaluated (A, B). Representative fluorescent intensity histograms (B, D) are shown and results are expressed as the mean of fluorescent intensity ± SEM of four independent assays and sample triplicates normalized to miR-negative control (NC) in A and corresponding miRNA in C. Red line-negative control (NC), blue line-miR-200c and purple line-miR-203 transfected cells (B) and blue line-miR-transfected cells, red line-miR-transfected cells treated with VP (D). * (*P* < 0.05) indicate statistically significant differences when compared the control group (NC)

Flow cytometry analysis of KCR cells transfected with miR-203, miR-200c and NC mimics allowed the quantification of the levels of DiOC_2_ accumulation in the presence and absence of verapamil, a known inhibitor of ABCB1 efflux pumps. As can be seen in Figure 6A, C, the activity of ABCB1 efflux pumps in mimic-miR-200c and mimic-miR-203 KCR transfected cells was significantly impaired compared to the control NC, confirming what we already demonstrated.

The ABCB1 efflux activity, 48 h after transfection with pre-miR-203 and pre-miR-200c, was verified by the capacity of transfected cells to retain DiOC_2_ in the presence of the efflux inhibitor VP [Figure 6C, D]. As expected, VP did not show a significant inhibitory effect in miR-203 and miR-200c transfected cells as shown in the representative histograms in Figure 6D (miR-transfected cells).

However, VP had a more pronounced inhibitory effect in the NC measured by an increased fluorescent retention inside the cells, which reflects functional efflux of ABCB1 efflux pumps. These results suggest that miR-transfected cells have reduced activity of ABCB1 efflux pumps, most probably and as expected derived from low expression/activity of the ABCB1, since more accumulation indicates less efflux.

### Expression of ABCB1/MDR1 in KCR transfected cells

The ABCB1 expression levels of both mRNA and proteins after cell transfection with miRNA-203 and miR-200c mimics were quantified by RT-qPCR and Western blot. There were no significant differences in ABCB1 expression levels between transfected cells and negative control.

**Figure 7:**
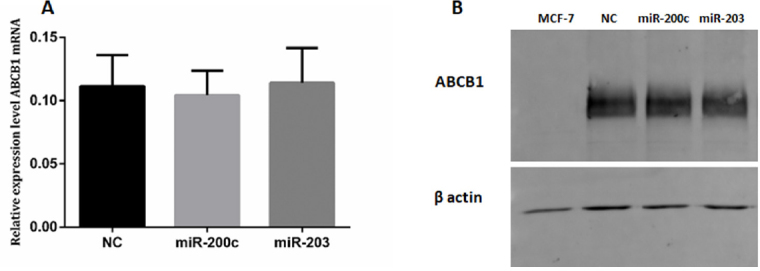
ABCB1 in KCR transfected cells. KCR cells were transfected with mimics-miRs (NC, miR-200c and miR-203) for 48 h and the levels of ABCB1 mRNA (A) was determined by RT-qPCR. miRNA expression was normalized to the house keeping gene GAPDH. Results are represented by mean values of three independent experiments and sample triplicates ± standard deviation. Protein expression (B) was analysed by Western blot analysis using β-actin as an internal control

Western Blot analysis of membrane proteins of MCF-7, Mimic-NC, miR-200c and 203 transfected cell using an anti-human ABCB1 monoclonal antibody (D-11). The 170 kDa band corresponds to ABCB1 protein [Figure 7].

## Discussion

The molecular mechanisms leading to drug resistance in cancer cells are wide. Chemotherapeutic drugs like doxorubicin and paclitaxel, described as ABCB1 substrates, can induce the up-regulation of ABCB1 efflux pumps during treatment, directing the upsurge of different acquired resistance mechanisms. The mechanisms underlying efflux pump overexpression still need to be elucidated before a strategy to overcome drug resistance can be found. However, it has been observed that this over-expression usually occurs as an early event in the development of MDR, which takes place before other mechanisms are established, such as mutations in drug targets, modification of drug metabolism, activation of DNA repair mechanisms or apoptotic death prevention^[38]^. Thus, an important early strategy to overcome MDR would be to target the overexpression of ABCB1.

In the present study, the modulating effect of miR-203 and miR-200c on the activity of efflux pumps in DOX resistant breast cancer cells that constitutively over-express ABCB1 efflux pumps, was investigated.

We have demonstrated that DOX resistant KCR cells, which over-express ABCB1 and present high levels of ABCB1 protein, have a low expression of miR-203 and miR-200c compared to the parental cell line MCF-7. These findings suggest a possible role for miR-203 and miR-200c in DOX resistance and consequently in the phenotype exhibited by KCR cells.

Recent studies demonstrated that miRNAs can directly or indirectly regulate ABCB1 gene expression^[18,39]^. In fact, bioinformatics’ analysis indicates that miR-200c and miR-203 are putative regulators of ABCB1, but only miR-200c has been experimentally validated^[23,39]^. Although miR-203 is known to have multiple targets, its role in drug resistance has been linked to different cancers. For example miR-203 have been linked to drug resistance in glioblastoma against imatinib, etoposide and temozolomide^[40]^, and in colorectal cancer against oxaliplatin^[41]^ and 5-fluorouracil^[42]^. In breast cancer miR-203 was associated to resistance to cisplatin^[25]^. miR-200c has been linked to resistance towards cisplatin in gastric cancer^[43]^, and paclitaxel in ovarian cancer^[44]^, besides doxorubicin^[45]^ and transtuzumab^[46]^ in breast cancer. Given the complexity in unravelling the circuitry of miRNA-mRNA interactions and the difficulty in validating targets, functional assays may be more important in the initial assessment of their role in modulating drug efflux.

Accordingly, to confirm the hypothesis of the potential role of miR-203 and miR-200c in drug resistance by over-expression of ABCB1, the efflux activity was assessed by cell uptake of the dye DiOC_2_, a specific substrate of ABCB1 transporter.

Our findings indicate that miR-203 and miR-200c have a negative role in the regulation of ABCB1 efflux pumps. This was reflected in the significantly higher capacity of miR-203 and miR-200c KCR transfected cells to retain DiOC_2_ when compared to control cells. The fact that ABCB1 mRNA levels were not significantly changed by transfection corroborated the hypothesis of post-transcriptional regulation of ABCB1 by miR-203 and miR-200c. Furthermore, using a specific efflux inhibitor of ABCB1 pumps, we were able to show that the increased intracellular accumulation of the dye was correlated with the interference caused by miR-203 and miR-200c in the transfected cells, expected to be due to a decrease in the number of functional ABCB1 efflux pumps units. However, Western Blot (WB) analysis did not reach a level to show significant differences in the expression levels of ABCB1 in both miR-203 and miR-200c transfected cells, raising the hypothesis that ABC expression levels do not always correlate with their functional activity, as previously reported in the literature^[47]^. The decrease of efflux activity after miRNAs transfection did not cause significant cell cytotoxicity after exposure to doxorubicin (data not shown), which seems in agreement with the western-blot data. A putative reduction in expression of ABCB1 may not be sufficient to increase cell viability within 48 hours. Doxorubicin can also be effluxed by different membrane-bound transporters besides ABCB1. On the other hand DiOC_2_ is a specific substrate for ABCB1, thus, it is possible that the concentration of microRNAs used in the transfection was not able to confer a significant result in the MTT viability assay as it was seen in the flow cytometry efflux assay as doxorubicin isn’t a specific substrate of ABCB1 as DiOC_2_^[48]^.

On the other hand, the significant overexpression of ABCB1 in KCR cells may not allow the assessment of a downregulation by these miRNAs by the techniques, protocols and time-frames used in this work. Katayama *et al*.^[49]^ demonstrated that the half-life of endogenously expressed ABCB1 is 26.7 ± 1.1 h in human colorectal cancer HCT-15 cells. Thus, the kinetics of downregulation of ABCB1 expression after transfection by pre-miRNAs may need a substantial period to reduce the levels of ABCB1 protein, more than the 48 h period chosen in this experimental setup.

It is also possible that WB analysis might not have been sensitive enough to distinguish small variations in the protein levels. If so, small changes in ABCB1 expression could be enough to reduce the efflux activity in order to be detected by the functional assays but not noticed by WB. Previous studies have reported differences in the expression level of ABCB1 protein after transfection with miR-200c, as miR-200c targets ABCB1 gene expression^[30]^. However, in the present study the methodological conditions were different. Differences include miRNA concentration used in the transfection, transfection agent, membrane extraction method and the origin of the resistant cells, hampering comparisons. In addition, we must be aware that working with high concentrations of miRNAs mimics can cause non-specific changes in gene expression of cells^[50]^.

However, as already described by Tomono *et al*.^[20]^, ABCB1 activity can be affected by cell membrane environmental changes. The miRNAs can target mRNA that code for proteins which interfere with the functionality of ABCB1. The altered expression levels of these miRNAs may be an early indicative of a possible development of drug resistance.

To the best of our knowledge, this is the first time that miR-203 and miR-200c are described as regulators of ABCB1 activity, either directly or indirectly. Nevertheless, the underlying mechanism responsible for the altered expression of both miR-203 and miR-200c and ABCB1 in doxorubicin resistance breast cancer and their specific role and mechanism of modulation of the activity of the ABCB1 efflux pump in drug resistance, still need to be elucidated.

## Declarations

### Copyright

© The Author(s) 2019.
